# Development of a functional impedimetric immunosensor for accurate detection of thyroid-stimulating hormone

**DOI:** 10.3906/kim-2012-69

**Published:** 2021-06-30

**Authors:** Engin ASAV

**Affiliations:** 1 Department of Nutrition and Dietetics, School of Health, Kırklareli University Turkey

**Keywords:** Thyroid-stimulating hormone, 4-mercaptophenylacetic acid, immunosensor, impedance spectroscopy, self-assembled monolayers

## Abstract

Thyroid-stimulating hormone (TSH), which regulates the synthesis of thyroid gland hormones affecting the whole metabolism, is a pituitary hormone. Determination of TSH is crucial for monitoring thyroid gland-related disorders and some metabolic diseases.In this study, a nonlabeled immunosensor based on covalent immobilization of anti-TSH antibody by using the formation of self-assembled monolayers (SAM) of 4-mercaptophenylacetic acid (4-MPA) and functionalization of carboxyl ends with 1-ethyl-3-(3-dimetilaminopropil) carbodiimide (EDC)/N-Hydroxysuccinimide (NHS) was fabricated for detection of TSH. Immobilization steps including the concentration of 4-MPA, the concentration of anti-TSH antibody, and duration of anti-TSH antibody incubation were optimized by utilizing electrochemical impedance spectroscopy. Under optimal conditions, a sensitive, rapid, and accurate determination of TSH at a concentration range between 0.7 and 3.5 mIU/L was accomplished with a notable linearity and LOD value of 0.034 mIU/L, as well as reproducibility and repeatability. Moreover, for comparison, linear range experiments were also carried out by using other electrochemical methods, including linear sweep voltammetry, cyclic voltammetry, and capacitance spectroscopy. Finally, the constructed immunosensor was used for analyzing TSH levels spiked in the artificial serum samples.

## 1. Introduction

A biosensor is described as “a compact analytical device incorporating a biological or biologically derived sensing element either integrated within or intimately associated with a physicochemical transducer” [1]. Since the first biosensor construction, various types of biological molecules including enzymes [2], antibodies [3], oligonucleotides [4], aptamers [5], tissue slides [6], and microorganisms [7] have been combined with a wide range of transducers such as amperometric [8], impedimetric [9], piezoelectric, acoustic [10], potentiometric [11], fluorescent [12] and colorimetric [13]. Antibodies have been abundantly used in the fabrication of biosensors named “immunosensors”, thanks to their ability to detect a specific antigen [14]. The method of electrochemical impedance spectroscopy (EIS) relies on measuring the impedance change caused by interactions between targets and bioreceptor [15]. The alterations on the electrode surface dramatically affect the signals obtained by EIS. Hence, EIS is widely used for monitoring antibody–antigen interactions in immunosensors along with nucleotide interactions on aptasensors or DNA biosensors [16]. Owing to their all-electrical nature, impedance biosensors are simpler than other methods used in biosensor construction. Moreover, since impedimetric transducers do not contain optical or acoustic components, they offer significant advantages for portable applications [17]. Immobilization of antibodies on a transducer capable of measuring little changes on the surface is a bottleneck to construct a stable and robust immunosensor, because of the orientation problem of antibodies and their conformational stability on the surface [18]. Several immobilization methods such as self-assembled monolayers (SAM) [19], electropolymerization [20], site-directed techniques [21], silanization [22], and direct covalent binding [23] have been utilized in the fabrication of immunosensors to handle these problems [24]. Immunosensors offer a number of advantages over traditional analytical techniques, including portability, specificity, cheapness, and real-time monitoring, as well as having good versatility, robustness, selectivity, and sensitivity depending on the transducer [25]. Hence, immunosensors have exhibited a significant development in the immune-analytical field in the last decade, which facilitates sensitive and accurate determination of a vast number of target molecules such as cancer biomarkers [26], pathogens [27], cardiac markers [28], drugs [29], pesticides [30], toxins [31], and hormone [32].

Thyroid-stimulating hormone (TSH), a.k.a thyrotropin synthesized and secreted by thyrotrophic cells in the anterior pituitary gland, stimulates the production and the secretion of the thyroxin (T4) and triiodothyronine (T3) in the thyroid gland [33]. Since T3 and T4 hormones are employed as mediators in several metabolic processes, TSH levels can affect the whole metabolism indirectly [34]. The normal TSH levels in adults are between 0.4 and 4.2 mIU/L[35]. Therefore, rapid, reliable, and sensitive detection of TSH levels is crucial for the diagnosis of thyroid gland-related diseases such as hypothyroidism, Hashimoto’s thyroiditis, Graves’ disease, and hyperthyroidism [36], as well as cardiovascular diseases, metabolic syndrome, pituitary tumors, atherosclerosis [37]. In the last 2 decades, determination of TSH levels can be accomplished by using various techniques such as tandem mass spectrometry [38], liquid chromatography/mass spectrometry [39], infrared spectroscopy [40], ultrafiltration [41], chemiluminescent enzyme immunoassay [42], and affinity assisted immunoassay [43]. Although some of these methods present the limit of detection (LOD) lower than 0.5 mIU/L, these techniques are troublesome, expensive, time-consuming, inappropriate for miniaturization, have tedious pretreatment steps and fabrication, demand sophisticated or heavy instruments, and require experienced personnel to operate [44]. These drawbacks can be overcome by immunosensors, which have characteristics including high sensitivity, specificity, and accuracy, as well as being relatively cheap and having a short response time [45]. Along with these benefits, immunosensors have some limitations, including regeneration problems, fragile antigen–antibody interactions, and low stability [46,47]. Nevertheless, a number of immunosensors with different transducers such as voltammetric [33,35], potentiometric [48], impedimetric [49], and fluorimetric [50] were reported for the detection of TSH. 

This study aims to develop an impedimetric immunosensor for determining TSH by using SAM of 4-mercaptophenylacetic acid (4-MPA) and a specific antibody of TSH named anti-TSH. Accurate and sensitive detection of TSH was accomplished by a nonlabeled immunosensor with a simple design. Furthermore, the proposed biosensor is the first biosensor that comparably performs TSH detection using four electroanalytical methods, including EIS, capacitance spectroscopy, linear sweep voltammetry (LSV), and cyclic voltammetry (CV). Additionally, experiments for repeatability, reproducibility, and standard addition in artificial human serum samples were also carried out. 

## 2. Materials and methods

### 2.1. Materials and reagents

1-ethyl-3-(3-dimetilaminopropil) carbodiimide (EDC), N-hydroxysuccinimide (NHS), 4-mercaptophenylacetic acid (4MPA), thyroid-stimulating hormone (TSH), thyroid-stimulating hormone antibody (anti-TSH), bovine serum albumin (BSA), and all the other chemicals were purchased from Sigma-Aldrich (St. Louis, MO, USA). In all experiments, preparation of solutions was carried out in certain solvents, including TSH and anti-TSH in ultrapure water (UPW), 4-mercaptophenylacetic acid (4-MPA) in absolute ethanol, as well as EDC, NHS, and BSA in 50 mM phosphate buffer at pH: 7.0. All dilutions and aliquots of TSH and anti-TSH were stored at −20 °C until use. A redox probe solution consisting of 5 mM Fe(CN)_6_^4−^ and 5 mM Fe(CN)_6_^3−^ was prepared in a 50 mM phosphate buffer system at pH: 7.0 that contained 0.1 M KCl as an electrolyte. The artificial serum solution was prepared in a 50 mM phosphate buffer system at pH: 7.5 by adding 2.5 mM urea, 0.1% human serum albumin, and 4.7 mM (D +)-glucose, as well as serum electrolytes including 4.5 mM KCl, 5 mM CaCl_2_, and 145 mM NaCl. The artificial serum solution was used without any dilution.

### 2.2. Instrumentation

All electrodes of the three-electrode system, including a gold working electrode, Pt wire as counter electrode, and Ag/AgCl as reference electrode, were purchased from BASi® Corporate (Indiana, USA). Ag/AgCl reference electrode was stored in 3 M KCl solution for saturation until usage. A PC-controlled device, Gamry Interface 1000, along with Echem Analyst^®^ software, which was used in all electrochemical experiments, was purchased from Gamry Instruments (Warminster, USA).

### 2.3 Construction of immunosensor

Before use, the Au electrodes were polished with alumina powder with a particle size less than 50 nm to obtain clear and smooth surfaces. Subsequently, Au electrodes were sonicated initially with absolute ethanol and then with deionized water for 10 min by using an ultrasonic bath to remove alumina particles and some probable chemical impurities. They were then dried with a pure argon gas stream. After the cleaning procedure, the bare electrode surfaces were examined by utilizing EIS spectra and Rct values.

**Figure 1:**
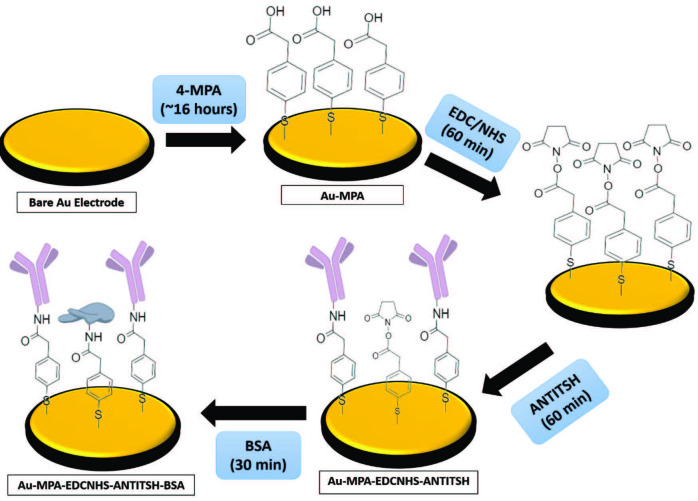
Immobilization steps of TSH immunosensors.

The immobilization method based on SAM of 4-mercaptophenylacetic acid was modified from Yağar et al. [51] and used for rapid and functional immobilization of anti-TSH antibody. Clean Au electrode was incubated in 4-MPA solution (10.0 mM, in absolute ethanol) for 16 h to constitute self-assembled monolayers onto the electrode surface. After this period, to remove unassembled 4-MPA molecules, the modified gold electrode was rinsed with ethanol and deionized water, then dried with a pure argon gas stream. Afterward, EDC and NHS reagents were employed for the functionalization of carboxyl groups on the modified electrode surface. For this purpose, a 10 µL aliquot of the EDC (0.2 M in PBS) – NHS (0.05 M in PBS) mixture was dripped onto the modified electrode, and then the electrode was incubated for 60 min in dark-moisture ambient. After rinsing with deionized water and drying process with pure argon gas of functionalized electrode, a 5 µL aliquot of anti-TSH (10 µg/mL) was applied onto the electrode surface and then incubated for 60 min in a moisture medium. After that, a 5 µL aliquot of BSA (0.1%) was dropped onto the antibody-modified electrode to cover up unbinding functional groups. Finally, to remove physically adsorbed proteins, the immunosensor was rinsed with deionized water and then gently dried with pure argon gas stream. Each step of immobilization is schematically represented in Figure 1.

Bare electrode and modified electrodes after each immobilization step are denoted in figures and tables as Bare Au, Au-MPA, Au-MPA-EDCNHS, Au-MPA-EDCNHS-ANTITSH, and Au-MPA-EDCNHS-ANTITSH-BSA, respectively. 

### 2.4. Principles of measurements 

Electrochemical impedance spectroscopy (EIS) was employed to determine TSH quantitatively and optimize and characterize modifications of electrode surface for each immobilization step. The solution of 5 mM Fe(CN)_6_^4−^and 5 mM Fe(CN)_6_^3−^, prepared in 50 mM PBS at pH:7.0 containing 0.1M KCl was served as a redox probe. For EIS studies, an alternating wave of 10 mV amplitude was applied to the electrode over the formal potential of the redox couple (0 V). Impedance spectra were collected in the frequency range between 10 and 50,000 Hz. 

**Figure 2:**
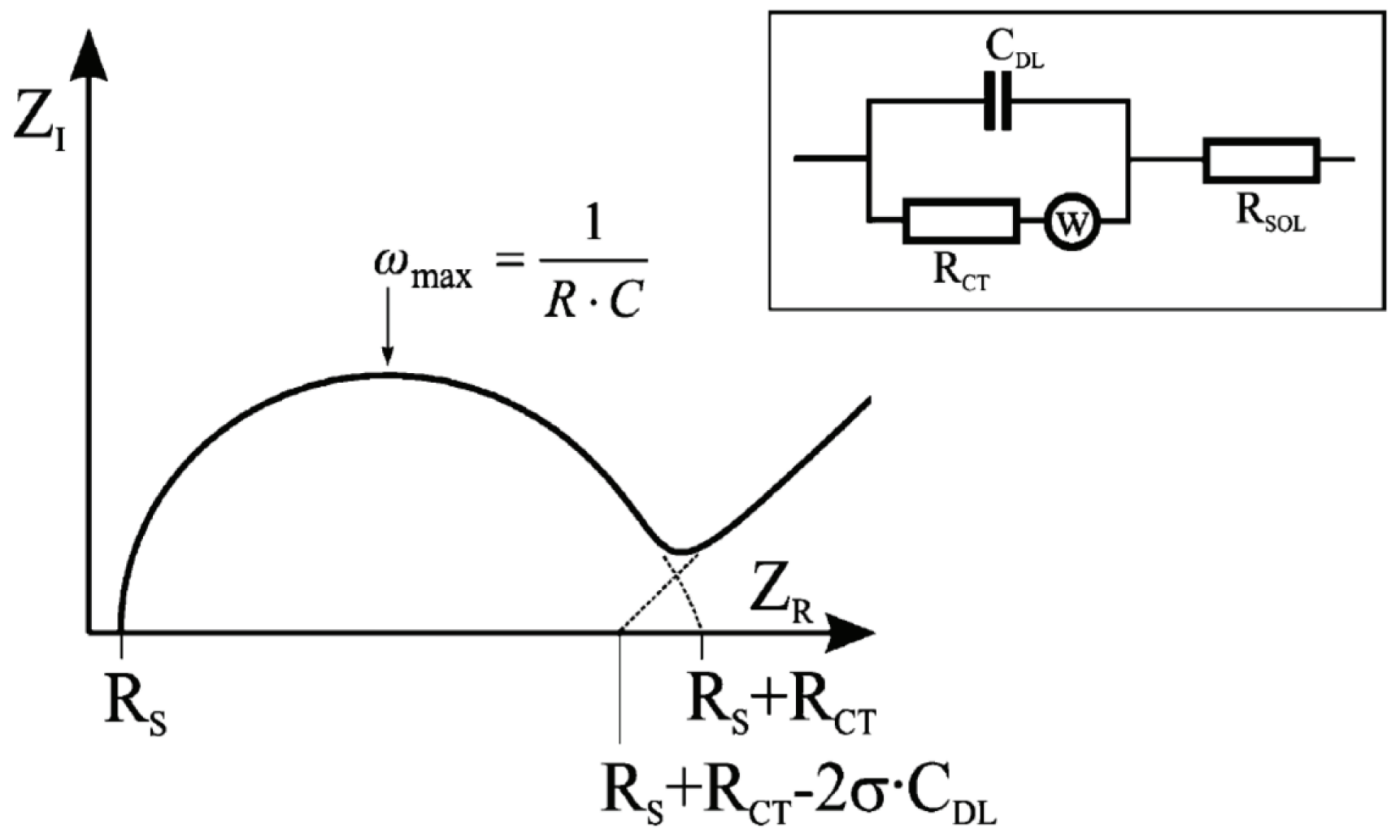
Randles equivalent circuit model for calculating Rct and capacitance.

The impedance signal is presented as a calculated function of the real and imaginary constituents (Zreal and Zim) in a Nyquist plot, which is shown in Figure 2. The linear part at lower frequencies corresponds to Warburg impedance, and the semicircle diameter at higher frequencies corresponds to the charge-transfer resistance (Rct), which can also be seen in Figure 2. To calculate Rct, Warburg elements and capacitance measurements were fitted on the software by using an equivalent circuit model. The equivalent circuit model in the present study also shown in Figure 2, named as Randles circuit, consists of an ohmic resistance (Rs) representing the resistance of the electrolyte solution, a double-layer capacitance related to the capacitive properties of the complex bioactive layer, a charge transfer resistance (Rct) along with a Warburg impedance (Zw), representing the diffusion of molecules to the electrode surface through the complex layer.

The data of EIS measurements were fitted with an equivalent circuit model, and then the calculated Rct values were collected easily by utilizing commercial software called Echem Analyst^®^ software. By using this software and equivalent circuit model, capacitance values could be determined by calculating the function between Rct and Warburg elements consisting of *Y* *_0_* and *alpha* values.

After each immunosensor construction, 5 µL aliquots of a standard TSH solution (0.7 mIU/L in ultrapure water) were dropped onto the modified electrode surface. Then the immunosensors were allowed to incubate in the same conditions for 60 min for each addition of standard TSH solution. After each incubation period, the immunosensors were rinsed with UPW to remove unbound TSH molecules from the immunosensor surface. Lastly, the immunosensors were placed into the electrochemical cell containing the redox probe solution. The electrochemical measurements were carried out as previously described above. The differences in charge transfer resistance values (ΔRct) between immunosensors unbound-bound TSH for each concentration were calculated for the preparation of TSH calibration curves.

LSV and CV were also utilized to detect TSH, for comparison of characteristics of calibration curves. LSV experiments in the same redox probe were carried out under the following conditions: potential range: –0.1 to 0.5 V, step size: 1.0 mV, scan rate: 50 mV/s. CV experiments with 3 cycles in the same redox probe were carried out under the following conditions: potential range: –0.1 to 0.5 V, step size: 1.0 mV, scan rate: 100 mV/s. Peak currents of the redox probe at the potential around 0.3 V calculated by Echem Analyst^® ^software were monitored and recorded for each method. Calibration curves were plotted by using differences of current values (ΔI) between baseline and TSH applied immunosensor for each concentration.

## 3. Results and discussion

### 3.1. Immobilization of anti-TSH onto Au electrode

All modifications of Au electrode surface, including the formation of 4-MPA self-assembled monolayers, functionalization of carboxyl groups via EDC/NHS reagents, and binding of anti-TSH antibody and BSA, were characterized using EIS. Nyquist plots of each immobilization step are shown in Figure 2. Rct values calculated by using these Nyquist plots for each step of the anti-TSH immobilization are given in Table 1.

**Table 1:** Rct values of each immobilization step.

Electrode	Rct value (ohms)
Bare Au	14.14
Au-4MPA	5490
Au-MPA-EDC/NHS	33.96
Au-MPA-EDC/NHS-ANTITSH	62.48
Au-MPA-EDC/NHS-ANTITSH-BSA	144.8

**Figure 3:**
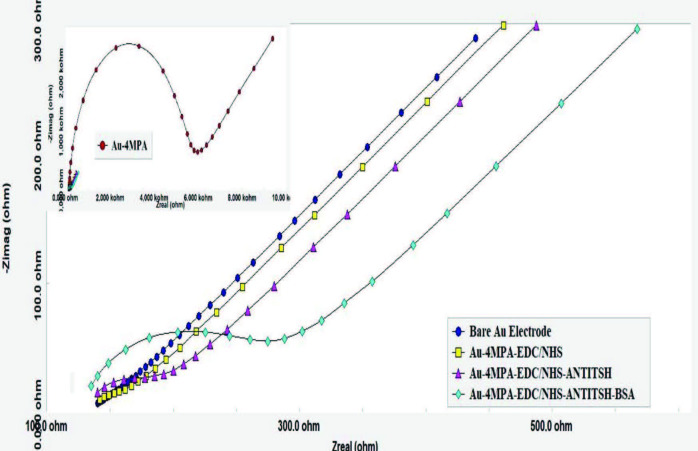
Nyquist diagrams of each immobilization step.

It is clearly seen in Figure 3 and Table 1 that the bare Au electrode showed a tiny semicircle diameter and Rct value, indicating a rapid transition of the redox probe to the surface. Furthermore, the increase of Warburg impedance indicating the diffusion of the redox probe through the electrode surface can be noticed for the bare electrode. The formation of 4-MPA monolayers on the electrode surface created a strict barrier to electron transfer that was revealed by neutral negative ends of the 4-MPA. Therefore, after modification with 4-MPA, the diameter of the semicircle portion and Rct values, as shown in Figure 3 and Table 1, increased dramatically. EIS responses for 4-MPA were similar to biosensors containing SAM of the other mercapto acids [52–54]. After the functionalization of carboxyl groups via the EDC/NHS reagents, an active ester as an intermediate occurred on the surface. Since it facilitates the diffusion of the redox probe to the surface, a dramatic decrease in Rct values was observed in Table 1. This is because of the generation of active ester as an intermediate, which could facilitate the diffusion of the redox probe to the surface. Additionally, this significant decrease shows that carboxyl groups of 4-MPA were successfully activated by EDC/NHS. EIS responses for EDC/NHS had similarities with previous studies in the literature [51,55–57]. Immobilization of anti-TSH antibody onto the functionalized electrode caused an increase in Rct values, as it might presumably block the transportation of the redox probe to the surface. Similarly, blocking of active ester intermediates via BSA increased charge resistance of the electrode surface.

### 3.2. Optimization of immunosensor 

Optimization experiments of the immobilization steps had great importance in evaluating effective detection characteristics for the immunosensor constructed. For this purpose, parameters including the concentration of 4-MPA, the concentration of anti-TSH antibody, and the duration of anti-TSH antibody incubation were optimized. Since functionalization of carboxyl groups via EDC/NHS with similar concentrations were studied and optimized before [51,57–59], optimization of the EDC/NHS concentration was not carried out. Moreover, optimum BSA concentration and duration of incubation were determined according to our previous work [57] and studies in the literature [60–63].

**Figure 4:**
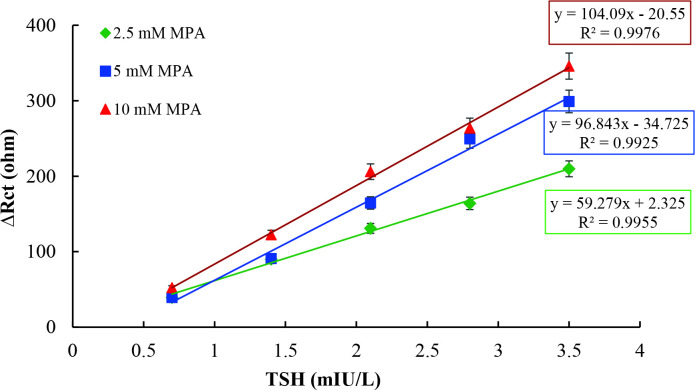
Calibration curves of immunosensor fabricated by using different 4-MPA concentrations [-♦-♦-(green): 2.5 mM 4-MPA, -■-■- (blue): 5.0 mM 4-MPA, -▲-▲-(red): 10 mM 4-MPA)].

The concentration of 4-MPA directly affected the detection ability of the immunosensor by changing the surface density of the gold electrode during the immobilization process. For the determination of optimum 4-MPA concentration, three immunosensors were fabricated by using 2.5 mM, 5 mM, and 10 mM 4-MPA solutions at the beginning of the immobilization process. The concentrations of all other chemicals used for the construction of immunosensor were kept constant. Calibration curves for each 4-MPA concentration, plotted between Rct values and TSH concentrations, are shown in Figure 4. 

**Figure 5:**
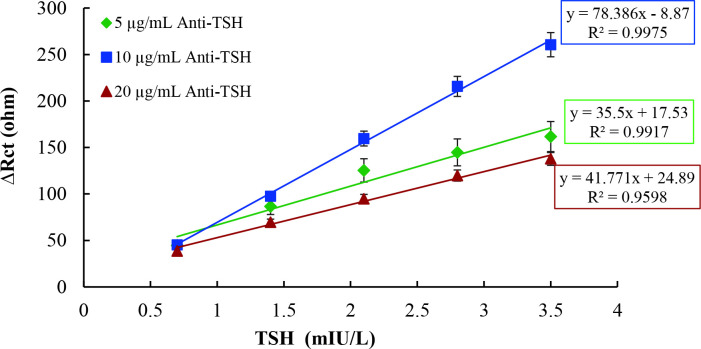
Optimization of concentration of anti-TSH antibody [-♦-♦-(green): 5 μg/mL anti-TSH, -■-■-(blue): 10 μg/mL anti- TSH, -▲-▲-(red): 20 μg/mL anti-TSH)].

It is obviously seen in Figure 4 that the increase in the 4-MPA concentration indirectly resulted in a gradual increase in signal rates of immunosensors. Enrichment of the amount of 4-MPA onto electrode surface elevates functionalization yield of EDC/NHS, which promotes anti-TSH antibodies for immobilization. Thus, the capability of TSH binding of immunosensor was increased dramatically. Therefore, the highest signal obtained immunosensor, which was constructed by using 10 mM 4-MPA, was selected as optimum for TSH detection. Furthermore, the coefficient of determination denoted as R^2^ of the proposed immunosensor, which shows the linearity of curves, was also the highest value. Moreover, the trend of decreasing sensitivity to TSH seen on graphics with decreasing 4-MPA concentration was inevitable. To determine the effects of anti-TSH amount binding to electrode surface on immunosensor response, both anti-TSH antibody concentration and duration of incubation of anti-TSH antibody were optimized. Firstly, to determine the optimum anti-TSH antibody, three immunosensors were fabricated by using different concentrations of anti-TSH, including 5µg/mL, 10µg/mL, and 20 µg/mL. Calibration curves for each immunosensor plotted between Rct values and TSH concentrations are shown in Figure 5.

**Figure 6:**
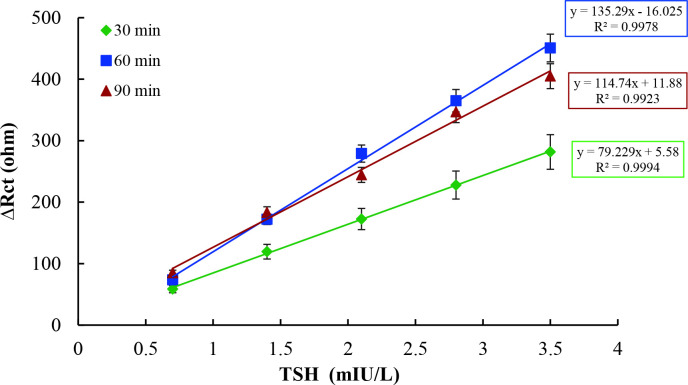
Optimization of incubation period of anti-TSH incubation [-♦-♦-(green):30 minutes, -■-■-(blue): 60 min, -▲-▲-(red): 90 min].

The sensitivity of an immunosensor depended on the amount of antibody; however, enhancing antibody amount on immunosensor surface does not always increase signal level and improve linearity. Due to orientation and positioning problems of antibody, blocking of electron transfer to the surface along with collapses and deformations on electroactive layer caused by the mass of antibodies, there is a saturation limit of immunosensor surface for each antibody. Hence, immunosensor constructed by using 20 µg/mL anti-TSH antibody had the worst results for both Rct values and linearity. As it can be clearly seen in Figure 5, although R^2^ values of immunosensors constructed by using 20 µg/mL and 10 µg/mL anti-TSH antibodies were similar, there was a significant difference between their signal levels. Thus, the optimum concentration of anti-TSH antibody was determined as 10 µg/mL for TSH immunosensor. Finally, functionalized electrodes were allowed incubation for three different periods, including 30 min, 60 min, and 90 min at the same anti-TSH concentration, to detect the optimum duration of antibody. Calibration curves for each period, plotted between Rct values and TSH concentrations, are shown in Figure 6.

As it can be easily seen in Figure 6, biosensor responses were significantly increased with the anti-TSH incubation period up to 60 min. Incubation periods shorter than 60 min as similar to the low concentration of anti-TSH antibody could not be sufficient for immobilization of anti-TSH onto the electrode surface. Therefore, it can be noticed that the binding of anti-TSH to the functionalized electrode surface could occur depending on the duration of incubation. Even though similar curves were obtained for incubation periods of 90 min and 60 min, there was a slight difference in R^2^ values representing accuracy. Additionally, a longer incubation time might cause possible biochemical interactions and deformations of the active layer that revealed a decrease in the stability and specificity of TSH immunosensor. Therefore, the optimum incubation period of anti-TSH antibody was determined as 60 min for TSH immunosensor construction. 

### 3.3. Analytical characteristics of TSH immunosensor

#### 3.3.1. Detection range of TSH immunosensor

The analytical characteristics of the proposed immunosensor, including detection range, LOD values along with repeatability and reproducibility, were evaluated for TSH. 

The detection range of TSH immunosensor was determined by using four different methods including EIS, LSV, CV, and capacitance. Electrode denoted Au-MPA-EDCNHS-ANTITSH-BSA was remarked baseline for all measurements, and calibration curves were plotted by calculating the difference in signals between baseline and TSH addition. These differences of signals calculated by utilizing Gamry Echem Analyst Software were remarked as ΔRct for impedance, ΔC for capacitance, and ΔI for CV and LSV on graphs.

**Figure 7:**
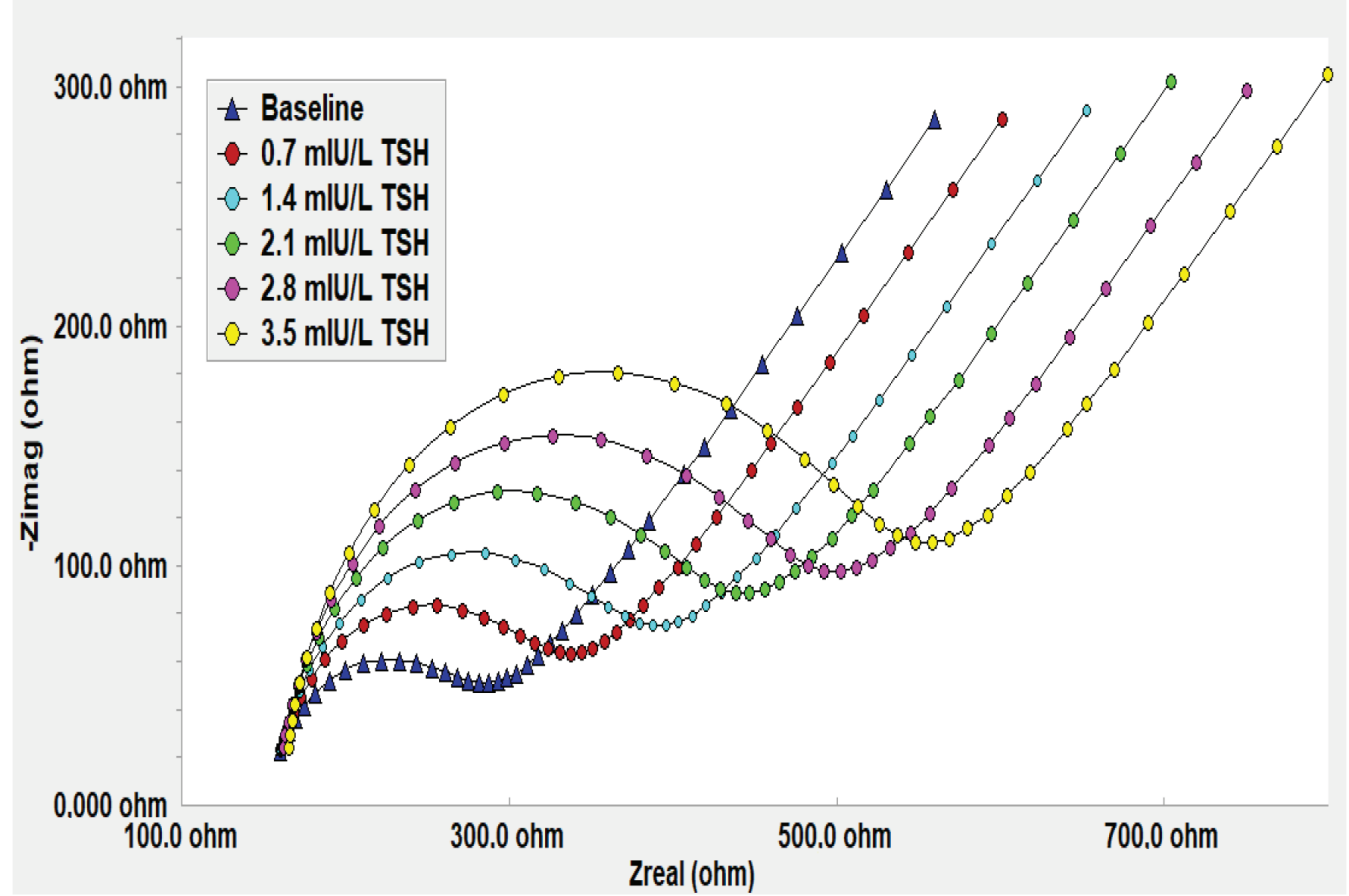
Impedance spectra obtained for different TSH concentrations.

**Figure 8:**
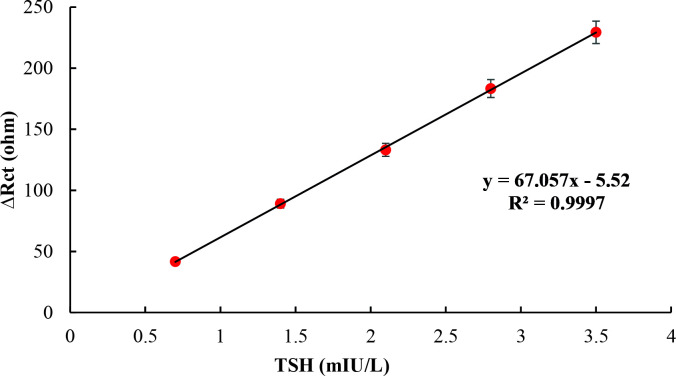
Calibration curve of TSH immunosensor obtained by using Rct values.

EIS responses of immunosensor obtained for different concentrations of TSH including 0.7, 1.4, 2.1, 2.8, and 3.5 mIU/L are shown in Figure 7. It is clearly seen in results that expanding semicircles of Nyquist plots regularly by addition of TSH resulted in a significant increase in Rct values. These characteristic Nyquist plots were similar to other EIS-based immunosensors [3,63,64], which were designed for the detection of protein-based analytes. By using Rct values calculated from these Nyquist plots, the calibration curve shown in Figure 8 was plotted for TSH at a concentration range of 0.7–3.5 mIU/L.

**Figure 9:**
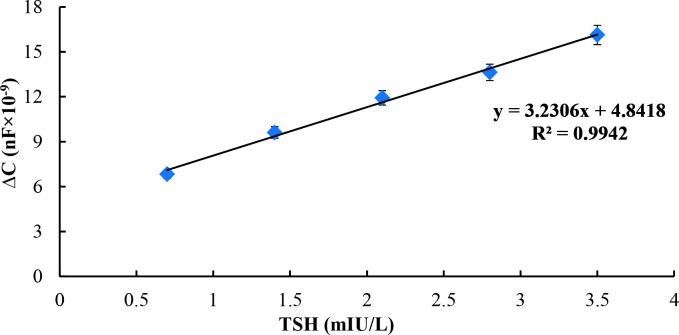
Calibration curve of TSH immunosensor obtained by using calculated capacitance values.

Additionally, another calibration curve for TSH, which is shown in Figure 9, was also prepared by using capacitance differences correlated with increasing TSH concentrations. Capacitance values were calculated for each concentration of TSH by using Rct, alpha, and Yo values of Nyquist plots are shown in Figure 7. Similarly, calculated capacitance values were used as a quantification method in a study by Limbut et al. [65] and our previous work [66]. 

**Figure 10:**
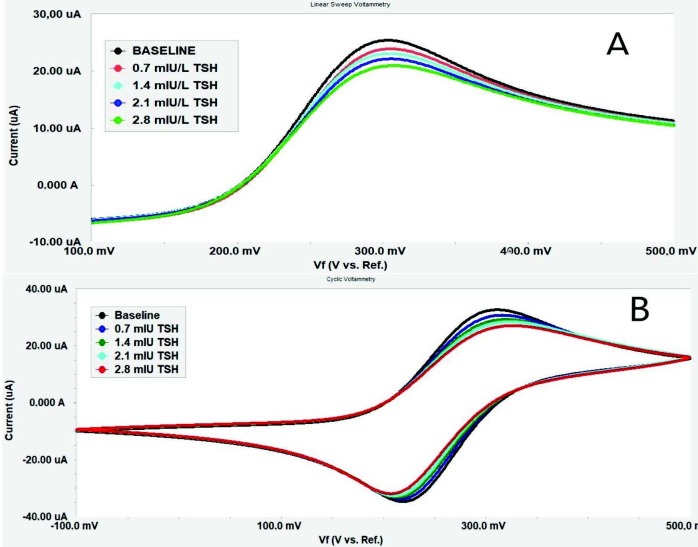
LSV (A) and CV (B) responses of TSH immunosensor

**Figure 11:**
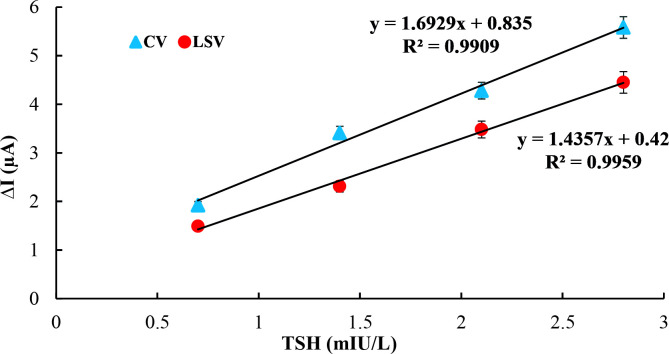
Calibration curves obtained by using LSV and CV responses of TSH immunosensor.

Immunosensor responses based on CV and LSV methods for TSH, including 0.7, 1.4, 2.1, and 2.8 mIU/L, are shown in Figure 10. As it is clearly seen in the results, the peak levels of both methods were in a tendency of prominent decrease against baseline by increasing concentrations of TSH. These results obtained for CV and LSV were similar to those of other immunosensors, which utilized CV or LSV as a quantification method to determine an analyte [67–71]. For each method, a calibration curve shown in Figure 11 at a linear range between 0.7 and 2.8 mIU/L was plotted by using peak currents generated automatically by EChem Analyst software. 

For each method, the limit of detection (LOD) representing the lowest detected quantity of TSH immunosensor was determined via the equation of *3.3Sd/m* . *Sd* and *m* , which represent the standard deviation of the intercepts and slope of the calibration curve, respectively, were calculated by using the regression module of Microsoft Excel^® ^software. LOD values obtained from data of calibration curve for LSV, CV, capacitance, and EIS were determined as 0.090 mIU/L, 0.134 mIU/L, 0.150 mIU/L, and 0.034 mIU/L, respectively. Since there was no study in the literature that utilized neither the LSV method nor the CV method for TSH determination, a comparison could not be made in terms of analytical parameters such as LOD, linearity, and linear range.

The present TSH immunosensor was compared to the other methods for TSH detection in such parameters including linear range, LOD, and linearity in Table 2.

**Table 2:** Comparison of present immunosensor to earlier TSH biosensors

Method	Principle of measurements	Detection range (mIU/L)	Linearity (R2)	LOD (mIU/L)	Ref.
PMMA nanobead labeled immunosensor	Fluorescence	0.05–100	0.9982	0.4	[50]
Immunosensor based on an azo compound	EIS	0.2–20.0	0.9960	0.04	[33]
Gold nanoparticle-based biosensor	Surface plasmon resonance	0.4–12.5	0.99	1.71	[72]
Lateral flow immunoassay	Raman spectroscopy	0–30	0.9946	0.025	[45]
Point of care device	Chemiluminescance	1.9–55	0.9942	1.9	[74]
Copolymer-based immunosensor	Potentiometry	1.45–17.5	N/A	1.4	[48]
ELISA membrane-based immunoassay	Differential pulse voltammetry	0.3–19.2	0.98	0.21	[73]
Nonlabeled immunosensor	EIS	0.7–3.5	0.9997	0.034	Present work

As it is obviously seen in Table 2, although all of the other methods [33,45,48,50,72–74] could detect TSH with a wide range, our nonlabeled TSH immunosensor showed better linearity than all of them. Moreover, in higher concentrations of TSH, the sample can easily be diluted and applied to the proposed TSH immunosensor. Additionally, methods obtaining LOD levels as low as the present biosensor had an expensive and complicated construction process as well as worse linearity. Since both linearity and LOD values are crucial parameters for precise and sensitive detection of an analyte, our present work is a successful example of an accurate immunosensor, which had a simple, cheap, and nonlabeled fabrication process. 

As clearly seen in the results, the detection range of the designed TSH immunosensor had good linearity along with decent LOD values for each method. Since EIS had better values for both of these parameters, it had been used only to determine characterization of other parameters such as reproducibility, repeatability, and performance on the artificial serum. 

#### 3.3.2. Reproducibility and repeatability 

Reproducibility representing the accuracy of the measurement method based on electrochemical immunosensors is one of the considerable parameters for biosensor construction. It means that the reproducibility expresses the difference between the two results of immunosensors, which are constructed by using the same parameters. For revealing reproducibility, ten TSH immunosensors were constructed under the same optimum conditions and calibration curves were obtained by using Rct values and TSH at concentration range of 0.7–3.5 mIU/L for each of these immunosensors. R^2^ values representing linearity and linear equations are given in Table 3.

**Table 3:** Reproducibility of TSH immunosensor.

Immunosensor	Linear equation (y=mx+n)	Linearity (R2)	Linear range (mIU/L)
1	y = 58.629x + 9.96	0.9995	0.7–3.5
2	y = 62.329x + 0.29	0.9948	0.7–3.5
3	y = 60.943x – 17.9	0.9959	0.7–3.5
4	y = 63.557x – 8.35	0.9948	0.7–3.5
5	y = 67.257x + 31.3	0.9919	0.7–3.5
6	y = 64.007x – 5.52	0.9997	0.7–3.5
7	y = 64.729x + 12.87	0.9996	0.7–3.5
8	y= 56.671x + 19.27	0.9956	0.7–3.5
9	y = 63.629x + 11.74	0.9948	0.7–3.5
10	y = 59.829x – 9.58	0.9908	0.7–3.5

Repeatability experiments were carried out to determine the average value, standard deviation, and coefficient of variation for TSH concentration at 0.7, 2.1, and 3.5 mIU/L. For this purpose, Rct values of immunosensors were recorded when the biosensor was consecutively exposed to aliquots of each 0.7, 2.1, and 3.5 mIU/L standard solutions on five occasions. The results for each level are shown in Table 4.

**Table 4:** Repeatability of TSH immunosensor

TSH concentration(mIU/L)	Average value (mIU/L)(n = 5)	Standard deviation (±) (mIU/L)(n = 5)	Coefficient of variation (%)(n = 5)
0.7	0.69	0.017	2.47
2.1	2.11	0.048	2.27
3.5	3.47	0.057	1.98

As it is obviously seen in Tables 3 and 4, our simply constructed and nonlabeled immunosensor had better repeatability and reproducibility than much of the other TSH immunosensors in the literature [33,35,45,49,50,75]. Additionally, the proximity of slope of curves, goodness of linearity along with lower standard deviation, and coefficient of variation have significant importance in developing an accurate immunosensor. 

#### 3.3.3. Application to artificial serum samples

Finally, TSH concentrations spiked in the artificial serum samples were determined by the immunosensor. The results presented in Table 5 show that the developed non-labeled immunosensor could precisely and accurately detect TSH in the artificial serum samples. Furthermore, these results also demonstrate that the proposed immunosensor was not affected by the interference of salts, urea, glucose and BSA.

**Table 5:** TSH detection in artificial serum samples.

Added TSH (mIU/L)	Detected by immunosensor (mIU/L)	Recovery rate (%)	Relative difference(%)
1	0.966	96.61	3.39
1.5	1.468	97.90	2.10
2.5	2.541	101.64	1.64
3	3.095	103.18	3.18

As shown in Table 5, our simply-fabricated TSH immunosensor showed similar performance in the artificial serum by the means of recovery rate and relative difference, compared to the previous biosensors in literature [3,55,76,77].

## 4. Conclusion

In this study, a sensitive, rapid, and accurate determination of TSH is accomplished by using a nonlabeled, low-cost, and simple-fabricated immunosensor. The proposed immunosensor shows good linearity in calibration curves, which is plotted by using values of four different electrochemical methods including EIS, LSV, CV, and capacitance for detection of TSH at a concentration range of 0.7–3.5 mIU/L. These methods are compared to each other in terms of linearity, detection range, and sensitivity. The proposed immunosensor has notable results in experiments of analytical parameters such as reproducibility and repeatability with low standard deviation and coefficient of variation along with LOD value as 0.034 mIU/L. Moreover, the constructed biosensor is also compared with some other TSH detection methods. As a result, the present immunosensor can be a cheap, accurate, sensitive, and easily constructed alternative to these methods. Furthermore, the proposed immunosensor does not consist of any heavy instruments, labeling process, and time-consuming pretreatment as in traditional methods for determination of TSH. Finally, detection of TSH spiked in the artificial serum is carried out successfully, and the results show that the fabricated immunosensor is a promising example for detection of TSH in serum samples. 
